# Bone mineral density in juvenile onset systemic lupus erythematosus from sultanate of oman

**DOI:** 10.1186/1546-0096-12-S1-P143

**Published:** 2014-09-17

**Authors:** Reem Abdwani, Eiman Abdulla, Saif Al Yaroubi, Haddia Bererhi, Ibrahim Al Zakwani

**Affiliations:** 1 Child Health, Sultan Qaboos University Hospital, Muscat, Oman; 2 Radiology, Sultan Qaboos University Hospital, Muscat, Oman; 3 Pharmacy, Sultan Qaboos University Hospital, Muscat, Oman

## Introduction

The survival of patients with systemic lupus erythematosus has increased over the last decade, which is associated with increased risk of morbidity. Osteoporosis remains one of leading morbidity associated with long term survivals. Multiple factors have been proposed to contribute to osteopenia, including limited physical activity, imited exposure to sunlight, duration of the disease activity, corticosteroid therapy, other immunosuppressant therapy, inadequate dietary intake of calcium and vitamins, and renal insufficiency. Children with Juvenile onset systemic lupus erythematosus (JSLE) are at even greater risk of developing osteopenia since the disease develops even before achieving their full potential peak bone mass.

## Objectives

We evaluated bone mineral density (BMD) in Omani children with JSLE in order to detect potential predictors of reduction in bone mass.

## Methods

BMD measurements were obtained in 27 JSLE patients and 97 healthy matched controls. The age, body mass index (BMI), BMD scores and Vitamin D levels were compared in both groups. Serial annual BMD was obtained in all JSLE patients and the first and last BMD scores were used for comparison. Factors that may play a role in BMD scores in the JSLE cohort including disease duration, disease activity, current steroid, cumulative steroid, immunosuppressive medication, vitamin D supplementation were recorded.

## Results

In comparing JSLE with healthy matched controls, patients with JSLE had lower BMI (16±2 versus 19±5 kg/m2 p value 0.007) which was associated with lower overall BMD than normal healthy cohort (0.72±09 versus 0.84±10 gm/cm 3 p value <0.001). It was interesting to note that the vitamin D level were higher in JSLE patients than normal healthy controls (61±17 versus 46±16 nmol/l p value <0.001).

When comparing serial BMD measurements in JSLE patients, the mean age at diagnosis was 6±3 years with mean disease duration of 4±3 years. All patients with JSLE had abnormal Z score on first BMD measurement; in addition between the first and last BMD scores there was a significant decline in Z scores measured -2.2±0.7 versus -2.6±0.7 ( p value 0.038). It was interesting to note that there was no change in overall BMD measurements during the study, while there was a significant increase in spinal BMD scores 0.46±0.09 versus 0.53±0.13 gm/cm3 with p value 0.002. Despite significant improvement in disease activity during the study, SLEDIA 16±7 versus 5±4 (p value 0.001), there was a significant decline in Z score. Other factors that may play a role in BMD measurements in JSLE patients, such as current and overall cumulative steroids and other immunosuppressive medication did not have a significant correlation with decline in BMD score in the study

## Conclusion

JSLE had lower BMI and BMD at disease onset than normal healthy cohort. Disease duration seems to play a major role in decline in Z score in the study. Other factors such as disease activity, vitamin D levels, current and cumulative steroids or immunosuppressive therapy did not seems to play a major role in decline in Z score. There was a significant increase in overall BMD spinal score in patients with JSLE which could be explained by effect of calcium and vitamin D supplementation. This study is not without limitation. A larger multi-centered study is needed to study the effect of JSLE on bone metabolism.

## Disclosure of interest

None declared.

